# Pork-cat syndrome?

**DOI:** 10.1186/2045-7022-5-S3-P164

**Published:** 2015-03-30

**Authors:** Carmo Abreu, Raquel Gomes, Bial-Arístegui Bartolome Borja, Helena Falcão, Leonor Cunha

**Affiliations:** 1 Immunoallergology Department, Centro Hospitalar do Porto, Porto, Portugal; 2 Immunoallergology Department, Centro Hospitalar Universitário de Coimbra, Coimbra, Portugal; 3 R&D Department, Bilbao, Spain

## 

Pork–cat syndrome was first described by Drouet et al, in 1994. It is a rare pathology, seen in patients sensitized to cat epithelium, which present symptoms suggestive of IgE-mediated hypersensitivity upon ingestion of pork meat. These symptoms range from urticaria with/without angioedema, to potentially fatal anaphylaxis. This rare syndrome is a result of a cross-reactivity of a protein of approximately 66kDa, identified as cat serum albumin (SA), which is highly homologous with porcine SA. In some patients, this cross-reactivity seems to extend to other mammals meat.

In this study, we aim to present a case of a 76 year-old patient, just known to be sensitized to cat epithelium, that immediately after the intake of rice and beef, started with generalized urticaria and aqueous, limited and non hematic diarrhea. He was assisted at the emergency department, with an excellent response to oral corticosteroid and antihistamines, without any residual damage.

After a two months period of totally avoiding red meat, the patient presented an anaphylatic shock, in an inadvertent ingestion of beef, pork and sausages at a barbecue. After being again assisted at the hospital he was referred for our immunoallergology department.

We performed - (1) skin prick tests to aeroallergens and commercial food extracts: positive to milk, cat and dog epithelium; (2) laboratory tests: blood count (normal); total IgE (559KUA/L - Phadia ImmunoCAP^®^) and specific IgE for cat epithelium (4.82KUA/L), dog epithelium (1.04KUA/L), milk (2.72KUA/L), beef (8.6KUA/L), pork (6.67KUA/L) and for cat serum albumin that revealed to be also positive. It was carried out a cow-milk oral challenge test with a cumulative dose of 200ml that was negative. SDS PAGE studies IgE-immunoblotting were also performed using extracts of pork, cow and cat epithelium that showed a common IgE binding band with a molecular weight of 67kDa.

This study describe a rare case of meat allergy with probably cross reactivity to a cat origin protein (primary sensitization), extended not only to pork meet but also to cow meat. This hypothesis is reinforced by the presence of a 67kDa protein which may correspond to the cat SA already described in the literature found in cow, pork and cat extracts. The patient is keeping red meat eviction diet (for 8 months now), and ever since, no reactions were reported.

## Consent

Written informed consent was obtained from the patient for publication of this abstract and any accompanying images. A copy of the written consent is available for review by the Editor of this journal.

